# Invasive Pneumococcal Disease Secondary to Acute Otitis Media in a High-Risk Patient: A Case Report and Review of Recent Changes to Pneumococcal Immunization Guidelines

**DOI:** 10.7759/cureus.28557

**Published:** 2022-08-29

**Authors:** Joshua Gregory, Nathaniel Chohrach, Deepa Iyengar

**Affiliations:** 1 Family and Community Medicine, The University of Texas, Health Science Center at Houston McGovern Medical School, Houston, USA

**Keywords:** pcv, acute otitis media, immunization, mastoiditis, meningitis, austrian triad, vaccine, pneumococcal, streptococcus pneumoniae, invasive pneumococcal disease

## Abstract

*Streptococcus pneumoniae* can commonly cause otitis media, sinusitis, pneumonia, or meningitis; however, these infections less frequently can develop into invasive pneumococcal disease (IPD). Vaccination for the prevention of pneumococcal disease has significantly decreased complications from severe infections, including pneumonia, meningitis, and IPD, in patients with certain risk factors. In this case study, we describe a unique presentation of disseminated *S. pneumoniae* meningitis and bacteremia in a patient who initially presented with acute otitis media (AOM). Due to the patient's multiple comorbidities of obesity, tobacco use, pre-diabetes, coronary artery disease, and lack of pneumococcal vaccination, their AOM rapidly progressed to life-threatening, an invasive pneumococcal infection which was successfully treated with timely initiation of antibiotics. In addition to discussing the patient's clinical course and treatment regimen, we will review pertinent updates to the pneumococcal vaccination guidelines for high-risk patients and their efficacy in preventing severe disease.

## Introduction

*Streptococcus *​​​​*pneumoniae* has become the most common cause of bacterial meningitis in the United States (US), accounting for 61% of total cases [1]. Most severe cases tend to develop in adults older than 65 years, children younger than five years, or patients with significant comorbidities [2]. In an effort to reduce the disease burden, the 23-valent vaccine known as pneumococcal polysaccharide vaccine 23 (PPSV23) was developed and implemented as the standard of care for several years. Previous studies isolating bacterial serotypes from cerebrospinal fluid (CSF) in patients with pneumococcal meningitis suggest 74-90% of these serotypes are contained in PPSV23 [1]. Pneumococcal conjugate vaccines (PCV), known as PCV15 and PCV20, are newer vaccines that have recently been recommended for use in adult populations. The appropriate and timely use of the newer pneumococcal vaccines can prevent severe pneumococcal infection among adults and children.

## Case presentation

A 61-year-old female with a past medical history of hypertension, coronary artery disease with three coronary artery bypass grafts, 30 pack-years of tobacco use, pre-diabetes, obesity, and no pneumococcal vaccination presented to the emergency room with acute encephalopathy, a Glasgow Coma Score (GCS) of 10, bloody discharge from her left ear and nose, and coffee-ground emesis. The previous day, she had been evaluated in the emergency room for a complaint of left ear pain. At that time, vital signs were normal, and an otoscopic exam revealed a normal right ear but left ear erythema with perforated tympanic membrane (TM). This led to a diagnosis of acute otitis media (AOM), and she was discharged home with a course of ofloxacin antibiotic ear drops for the left ear. Now, vital signs were significant for a fever of 103.3 F and hypertension of 238/112 mmHg. Physical examination revealed neck stiffness, perforated TM on the left side, and minimal responsiveness except to painful stimuli. The rest of the exam, including cardiac auscultation, was unremarkable though limited due to her encephalopathy. Laboratory studies showed electrolytes within normal ranges, elevated inflammatory markers including lactic acid and procalcitonin, and hyperglycemia of 225 mg/dL. Furthermore, complete blood count (CBC) was significant for leukocytosis of 12.5×10^3^/µL with an elevated absolute neutrophil count of 10.9×10^3^/µL. Due to clinical suspicion of meningitis, lumbar puncture (LP) was performed, with findings seen in Table 1.

**Table 1:** Cerebrospinal fluid analysis from lumbar puncture NAAT - nucleic acid amplification testing; CSF - cerebrospinal fluid

Appearance (clear)	Cell count and differential (<5 mm^3^)	Opening pressure (5-18 mmHg)	Red blood cells (0 mm^3^)	Protein (15-45 mg/dL)	Glucose (40-75 mg/dL)	*S. pneumoniae* NAAT CSF
Cloudy with xanthochromia	650 mm^3^ (79% neutrophils)	29 mmHg	2,320 mm^3^	919 mg/dL	<1 mg/dL	Detected

Due to concern for elevated intracranial pressures from increased opening pressure on LP, a fundoscopic exam was performed, showing no disc edema. Computed tomography (CT) of the head with and without contrast revealed left mastoid effusion and maxillary sinusitis, chest radiograph (CXR) was negative for pneumonia or infectious process, magnetic resonance imaging (MRI) of the brain with venous sinuses revealed no thrombus but had changes consistent with chronic hypertension, and electroencephalogram (EEG) revealed mild diffuse encephalopathy with no epileptiform activity. Empiric antibiotic therapy was started with cefepime and vancomycin and was broadened with ampicillin and acyclovir on day zero. Empiric steroid therapy was discussed but not initiated as the appropriate time frame for administration had passed.

**Figure 1:**
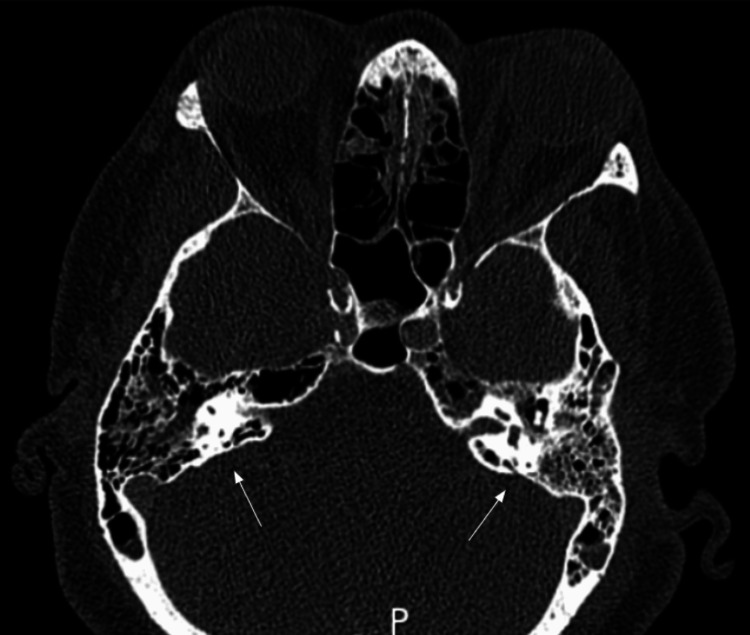
Partially opacified bilateral mastoid air cells and middle ear cavities, consistent with mastoiditis

Initial blood cultures were positive for *S. pneumoniae* on day zero. Upon receiving final culture sensitivities on day one, antibiotic therapy was narrowed to ceftriaxone and vancomycin. *S. pneumoniae* was later detected in the original CSF collection via nucleic acid amplification testing (NAAT). CT sinus/temporal bone was obtained on day two of hospitalization, which showed paranasal mucoperiosteal sinus thickening and bilateral opacified mastoid air cells consistent with mastoiditis (Figure 1). Bilateral myringotomy tubes were placed on day three of hospitalization, and a two-week course of bilateral ofloxacin ear drops was initiated.

The patient experienced a significant clinical improvement in mental status and neck stiffness beginning on day three of hospitalization, along with a progressive resolution of her fever and leukocytosis. She was fully alert and oriented to name, place, date, and situation by day five and began working with physical and occupational therapy. The antibiotic regimen was de-escalated from ceftriaxone and vancomycin to a four-week course of intravenous (IV) ceftriaxone on day five. After the successful placement of a peripherally inserted central catheter (PICC) to receive IV antibiotics in the home setting, she was discharged on day eight of hospitalization with plans for further home health rehabilitation.

## Discussion

Invasive pneumococcal disease (IPD), defined as pneumococcal invasion in a previously sterile location, such as blood, cerebrospinal fluid, or pleural fluid, is a life-threatening complication of *S. pneumoniae* infection with an estimated prevalence of 10.6/100,000 in the US population [3]. There is an estimated 10% and 15% risk of mortality for pneumococcal meningitis and bacteremia, respectively, though the true disease burden of IPD is thought to be underestimated [3]. Compared to IPD, the incidence of community-acquired pneumonia (CAP) requiring hospitalization in the US population is 270/100,000 [4]. Data is limited when considering the occasional progression of other conditions such as pneumococcal sinusitis and otitis media; therefore, the development of IPD is likely even more uncommon than the data suggests. When using CAP as a marker for vaccine efficacy in overall pneumococcal disease, the prevalence of hospitalization for CAP declined by 54.8/100,000 in the US population since the initiation of pneumococcal vaccine regimens [4]. While antibiotics have been historically successful at treating pneumococcal disease and its complications, rising antibiotic resistance has led to a push to continue developing effective vaccines to prevent these infections.

Acute otitis media (AOM) can be defined as an infection of the middle ear space, with *S. pneumoniae* as the most common responsible pathogen [5]. Though AOM is commonly viewed as a childhood illness, it also occurs in adults and most commonly presents as otalgia with an inflamed or bulging tympanic membrane (TM) seen on an otoscopic exam. Additionally, adults who use tobacco products have a higher risk of middle ear pathology such as AOM, likely secondary to decreased ciliary and immune functioning [6]. A recent study investigating the disease burden of AOM in adult populations 15 years of age and older estimated the incidence at 5.3/1000 person-years, which remained stable between 2015-2018 [7]. Furthermore, the incidence of AOM was noted to decline with age, as individuals 64 years and older developed AOM at 2.7/1000 person-years [7]. The mainstay of treatment in the US for both pediatric and adult populations has been oral antibiotics such as high-dose amoxicillin or second-generation cephalosporins. In individuals with a TM perforation, such as our patient, ototopical antibiotics such as ofloxacin ear drops can be used instead of oral antibiotics due to their efficacy in delivering effective doses of antibiotics without systemic side effects [5].

Though our patient initially presented with AOM and TM perforation, initiation of ofloxacin ear drops was insufficient in preventing disease progression to IPD in the form of mastoiditis, meningitis, and bacteremia. Our case study is one of the first to document this rapid development from AOM to IPD. It is imperative for physicians to counsel adult patients with AOM in the primary care setting who have comorbid conditions such as diabetes, tobacco use, and lack of pneumococcal vaccination with strict return precautions and proper medication administration. Though our patient received the recommended treatment of ofloxacin ear drops, she potentially would have benefited from oral antibiotics due to her comorbidities and the possible presence of subclinical AOM on the right ear. This suspicion comes from the evidence of bilateral mastoiditis seen on her CT sinus/temporal bone during her hospitalization. 

One further consideration when evaluating a patient for possible IPD is a group of disease processes known as the Austrian triad. This is a rare triad of meningitis, pneumonia, and endocarditis, all presenting simultaneously from a severe *S. pneumoniae* infection. This triad is considerably rare and is more likely to present in males with alcohol use syndrome, with an associated mortality rate of 75% [8]. Though rare, the Austrian triad is important to consider when working up IPD. In our patient, no evidence of pneumonia or endocarditis was seen on imaging, given the unremarkable CXR and transthoracic echocardiogram (TTE). Furthermore, the patient had normal auscultation findings with no murmurs or cardiac-specific symptoms. However, given the severity of our patient's presentation, it is important for future physicians to consider clinical work-up for this triad in similar situations. 

Though the Austrian triad is an interesting and specific presentation of IPD, the disease process can vary greatly depending on the patient population, risk factors, and infectious source. The following Table 2 summarizes several additional case reports detailing other known presentations, treatments, and outcomes of IPD in adults [8-11].

**Table 2:** IPD manifestations and outcomes as documented by other published case reports IPD - invasive pneumococcal disease; PPSV23 - pneumococcal polysaccharide vaccine 23; PCV13 - pneumococcal conjugate vaccine 13; AOM - acute otitis media; TM - tympanic membrane

Age + sex	Risk factors + comorbidities	Vaccination status	Background + initial presentation	IPD manifestation	Antibiotic regimen	Outcome
61-year-old female (our patient)	Hypertension, coronary artery disease, tobacco use, pre-diabetes, obesity	No vaccination	Left ear pain, AOM with TM rupture	Meningitis, mastoiditis, bacteremia	Ceftriaxone, vancomycin, ofloxacin ear drops	The patient discharged on day eight of hospitalization
76-year-old female [8]	Diabetes mellitus, hypertension	Partially vaccinated (PPSV23 administered, lacking PCV13)	Headache, encephalopathy	Pneumonia, endocarditis, meningitis	Ceftriaxone, rifampin	The patient discharged on day 31 of hospitalization
76-year-old male [9]	Hypertension, laryngeal cancer, chronic obstructive pulmonary disease (COPD), diabetes mellitus	Unknown	Fevers, coarse crackles, wheezes in the right lung, and decline in oxygenation on day 66 of hospitalization for cerebral arteriovenous malformation hemorrhage with subsequent surgical hematoma evacuation	Hospital-acquired pneumonia, bacteremia	Piperacillin/tazobactam, teicoplanin	Patient expired
68-year-old female [10]	None	No vaccination	Subdural hematoma in setting of traumatic fall	Subdural empyema, meningitis, pneumonia	Meropenem	Patient expired
58-year-old male [11]	Pulmonary tuberculosis	Unknown	Fever, myalgias, and productive cough in the setting of SARS-CoV-2 exposure	Pneumonia, bacteremia	Ceftriaxone, azithromycin	Patient expired

To help decrease the prevalence of IPD and its various complications, the PPSV23 vaccine was developed as a capsular polysaccharide vaccine that was initially approved in 1983. PPSV23 was shown to decrease vaccine-type IPD in immunocompetent adult patients by 65%, with 80% of adults developing sufficient antibodies within two to three weeks of administration. However, PPSV23 was shown to have an insufficient immune response in children, specifically those under two years of age, likely due to the inability of young children to form T cell-independent immune responses, which this vaccine relies upon [12]. To this end, the PCV7 conjugate vaccine was developed in 2000 for use in pediatric populations, which led to an almost 70% decrease in infection caused by PCV7 serotype bacteria [13]. It also led to decreases in hospitalizations caused by pneumococcal pneumonia, likely secondary to decreased overall carrier rates in both children and adults [14]. A second conjugate vaccine called PCV13 was developed in 2010, which further contributed to decreases in invasive and noninvasive pneumococcal disease in pediatric and adult populations, including a 46% decrease in pneumonia and a 75% decrease in IPD [15]. 

For the last several years, PPSV23 and PCV13 have been the standards of pneumococcal vaccination. However, in early 2022, two new conjugate vaccines, known as PCV 15 and PCV 20, were approved for use. These vaccines include up to seven pneumococcal serotypes not previously covered by PCV13. The efficacy of these new vaccines was inferred by comparing antibody responses in vaccine-naive adults between the previously studied PCV13 and the new PCV20 through a process known as immunobridging. Though data is not yet available about the specific efficacies of the PCV15 and PCV20 vaccines, comparable efficacies of the formerly recommended PCV13 vaccine show 75% effectiveness against vaccine-type IPD and 46% effectiveness against vaccine-type pneumococcal pneumonia [15]. Given the high degree of certainty of the efficacy of these new vaccines, new guidelines for their use in adults have been established by the Advisory Committee on Immunization Practices (ACIP). 

For all adults 65 years and older who have never received a pneumococcal vaccine, the Centers for Disease Control's (CDC) updated guidelines recommend vaccination with either PCV15 followed by PPSV23 within one year or PCV20 with no subsequent PPSV23 dose needed. If the patient has received PPSV23 in the past, one dose of either PCV15 or PCV20 may be used at least one year after the previous PPSV23 dose, with no further doses needed. Finally, if the patient has received PCV13 in the past, one dose of PPSV23 should be given, as the efficacy of the newer PCV15 and PCV20 has not been evaluated in this population [16]. These recommendations are summarized in Table 3.

**Table 3:** Updated pneumococcal vaccination guidelines per CDC (2022) PPSV23 - pneumococcal polysaccharide vaccine 23; PCV13 - pneumococcal conjugate vaccine 13

Vaccination history	No pneumococcal vaccines received	PPSV23 received at least one year prior	PCV13 received at least one year prior
Updated recommendations (2022)	One dose of PCV15 followed by PPSV23 within one year, or one dose of PCV20	One dose of PCV15 or PCV20	One dose of PPSV23

Adults ranging from 19 to 64 years of age with certain underlying medical conditions or other risk factors who have not received a pneumococcal conjugate vaccine or whose previous vaccination history is unknown should receive PCV20 with no subsequent dose or PCV15 followed by PPSV23 [16]. A list of indicated underlying medical conditions or risk factors that necessitate vaccination in adults 64 years and younger includes but is not limited to alcoholism, chronic heart, liver, or lung disease, tobacco use, diabetes mellitus, chronic renal failure, nephrotic syndrome, immunodeficiency, iatrogenic immunosuppression, generalized malignancy, human immunodeficiency virus (HIV), Hodgkin's disease, leukemia, lymphoma, multiple myeloma, solid organ transplants, congenital or acquired asplenia, sickle cell disease or hemoglobinopathies, CSF leak, or cochlear implants [17]. 

Despite the presence of these effective tools for preventing pneumococcal disease, the actual vaccination rates in the community are not optimal. In 2020, the rate of pneumococcal vaccination among adults over 65 was 67.5%. However, the rate of vaccination in high-risk adults ranging from 19 to 64 years of age was significantly lower at 23.9%, with both vaccination rates slowly increasing since 2010. Of note, in both of these populations, minority adults had lower vaccination rates compared to white adults [18]. It is also important to note that this data examined vaccination rates before the introduction of the PCV15 and PCV20 vaccines.

## Conclusions

Despite the overall reduction in the rates of invasive pneumococcal disease due to the administration of pneumococcal vaccinations, our case details a life-threatening presentation of IPD in the form of meningitis and bacteremia from an initial presentation of AOM in an unvaccinated patient. The course and outcome in our patient both reinforce the importance of timely identification of IPD with its clinical presentation and the need for prompt initiation of antibiotics to prevent long-term morbidity and mortality. Despite the presence of efficacious pneumococcal vaccinations, the community rates of vaccination are suboptimal. Though this patient had several established risk factors for IPD, she had not received any pneumococcal vaccinations. The favorable outcome in our case provides some reassurance that if promptly identified and treated, recovery without significant morbidity is possible. Our discussion also highlights the established deficit that exists in vaccination rates in appropriate patients to prevent IPD, especially in patients 64 years and younger with known comorbidities. Since IPD is a lethal disease, it is imperative to reduce the disease burden through the universal dissemination and aggressive adoption of the updated recommendations for the newer pneumococcal vaccines. PCV15 and PCV20 are the newly recommended vaccines in addition to PPSV23 and PCV13, which are highly effective against most *S. pneumoniae* serotypes that cause IPD. Our case brings forth the need for us as health care providers to work together to reduce the morbidity and mortality related to IPD by practicing preventive medicine.

## References

[1] Brouwer MC, Tunkel AR, van de Beek D (2010). Epidemiology, diagnosis, and antimicrobial treatment of acute bacterial meningitis. Clin Microbiol Rev.

[2] (2022). Pneumococcal disease: surveillance, reporting and trends. https://www.cdc.gov/pneumococcal/surveillance.html.

[3] Berical AC, Harris D, Dela Cruz CS, Possick JD (2016). Pneumococcal vaccination strategies. An update and perspective. Ann Am Thorac Soc.

[4] Drijkoningen JJ, Rohde GG (2014). Pneumococcal infection in adults: burden of disease. Clin Microbiol Infect.

[5] Danishyar A, Ashurst JV (2022). Acute otitis media. https://www.ncbi.nlm.nih.gov/books/NBK470332/.

[6] Gaur K, Kasliwal N, Gupta R (2012). Association of smoking or tobacco use with ear diseases among men: a retrospective study. Tob Induc Dis.

[7] Rijk MH, Hullegie S, Schilder AG, Kortekaas MF, Damoiseaux RA, Verheij TJ, Venekamp RP (2021). Incidence and management of acute otitis media in adults: a primary care-based cohort study. Fam Pract.

[8] Rakočević R, Shapouran S, Pergament KM (2019). Austrian syndrome - a devastating Osler's triad: case report and literature review. Cureus.

[9] Sugimoto N, Yamagishi Y, Hirai J (2017). Invasive pneumococcal disease caused by mucoid serotype 3 Streptococcus pneumoniae: a case report and literature review. BMC Res Notes.

[10] Kagami H, Muto J, Nakatukasa M, Inamasu J (2011). Infected acute subdural hematoma associated with invasive pneumococcal disease. Neurol Med Chir.

[11] Ayad S, Alyacoub R, Gergis K, Grossman D, Salamera J (2021). Invasive pneumococcal disease in a patient with COVID-19: a case report. Cureus.

[12] (2022). Chapter 2: Epidemiology of meningitis caused by Neisseria meningitidis, Streptococcus pneumoniae, and Haemophilus influenza. https://www.cdc.gov/meningitis/lab-manual/chpt02-epi.html.

[13] Richter SS, Heilmann KP, Dohrn CL, Riahi F, Diekema DJ, Doern GV (2013). Pneumococcal serotypes before and after introduction of conjugate vaccines, United States, 1999-2011. Emerg Infect Dis.

[14] Daniels CC, Rogers PD, Shelton CM (2016). A review of pneumococcal vaccines: current polysaccharide vaccine recommendations and future protein antigens. J Pediatr Pharmacol Ther.

[15] (2022). About pneumococcal vaccines. https://www.cdc.gov/vaccines/vpd/pneumo/hcp/about-vaccine.html.

[16] (2022). Pneumococcal vaccine recommendations. https://www.cdc.gov/vaccines/vpd/pneumo/hcp/recommendations.html.

[17] (2022). Pneumococcal vaccination: summary of who and when to vaccinate. https://www.cdc.gov/vaccines/vpd/pneumo/hcp/who-when-to-vaccinate.html.

[18] (2022). Vaccination coverage among adults in the United States, National Health Interview Survey, 2019-2020. https://www.cdc.gov/vaccines/imz-managers/coverage/adultvaxview/pubs-resources/vaccination-coverage-adults-2019-2020.html#:~:text=Pneumococcal%20Vaccination&text=Coverage%20among%20adults%20aged%20%E2%89%A565%20years%20was%2067.5%25%2C%20similar,and%20Asian%20(54.9%25)%20adults.

